# Motivational Interviewing for Pediatric Residents: A Case-Based Workshop on Adolescent Substance Use

**DOI:** 10.15766/mep_2374-8265.11641

**Published:** 2026-10-22

**Authors:** Roshni Bhat, Natalie LaBossier, Adam Kronish, Kevin Nguyen

**Affiliations:** 1 Resident, Department of Pediatrics, The Children's Hospital of Philadelphia; 2 Resident, Department of Pediatrics, The Children's Hospital of Philadelphia; 3 Instructor, Division of Adolescent and Young Adult Medicine, Mass General Brigham for Children; 4 Attending Psychiatrist, Behavioral Health Integrated Program, The Children's Hospital of Philadelphia

**Keywords:** Motivational Interviewing, Substance Use Disorders, Adolescent Medicine, Addiction Medicine, Case-Based Learning, Pediatrics, Integrated Behavioral Health

## Abstract

**Introduction:**

Substance use counseling is an essential component of pediatric training, especially given the recent rise in substance use–related morbidity and mortality among adolescents. The Substance Abuse and Mental Health Services Administration (SAMHSA) and the American Academy of Pediatrics (AAP) recommend that clinicians and others treating youth incorporate screening, brief intervention, and referral to treatment (SBIRT) practices. Limited familiarity remains a barrier to implementation. This workshop aims to strengthen familiarity with motivational interviewing (MI) through interactive learning.

**Methods:**

This 90-minute session was conducted for second-year pediatric residents and was facilitated by pediatricians. The session included a 20-minute didactic about MI, 2 role-play cases, and structured debriefs. Materials included a facilitator guide, slides, an observer checklist, and MI reference sheets. Participants completed pre- and postsession surveys that assessed their self-reported knowledge, comfort, and intent to apply MI skills.

**Results:**

Twenty-four PGY-2 residents completed the initial survey, and 17 completed both pre- and postintervention surveys. Most had prior exposure to MI through lectures (92%), standardized patient encounters (79%), and case-based learning (75%). All agreed that MI training is crucial to their role. Among paired respondents, self-reported comfort increased in using MI, counseling patients, and referring patients to treatment. This intervention also increased knowledge scores.

**Discussion:**

This workshop improved residents’ self-reported comfort in applying MI during clinical encounters. The provided materials make the session adaptable for other residency programs.

## Educational Objectives

By the end of this activity, learners will be able to:

1. Identify fundamental principles of motivational interviewing for addressing substance use in adolescents.
2. Differentiate levels of adolescent substance use, including nonproblematic substance use and substance use disorder.
3. Demonstrate their comfort with using motivational interviewing to address adolescents with substance use.

## Introduction

Substance use counseling is of growing importance in pediatric training given the recent rise in substance use–related morbidity and mortality in the pediatric population.^1,2^ Clinicians and others treating youth are particularly important in the early identification and intervention of substance use that may develop into a disorder.^3,4^ In a nationally representative sample, the prevalence of substance use disorders (SUDs) was higher in adolescents within 12 months of initiation of cannabis or prescription drug misuse when compared to young adults aged 18 to 25 years. This is consistent with studies demonstrating that an earlier age of initiation of use is a risk factor for the development of an SUD.^5^

The Substance Abuse and Mental Health Services Administration (SAMHSA) recommends universal screening for adolescent substance use and SUD by applying the screening, brief intervention, and referral to treatment (SBIRT) framework as part of routine health care.^6,7^ The American Academy of Pediatrics (AAP) recommends that pediatricians increase their capacity in substance use detection, assessment, and intervention. Pediatricians should become familiar with adolescent SBIRT practices and their potential to be incorporated into universal screening and comprehensive care of adolescents.^8^ Yet, pediatricians report significant barriers to implementation, with a lack of clinical familiarity being among the most common.^9^ Although the importance of substance use education and SBIRT practice is widely recognized, prior work has demonstrated variability in the implementation of such training across health professional training programs.^10^

Recent Accreditation Council for Graduate Medical Education (ACGME) changes for pediatric residencies, which took effect on July 1, 2025, include a new 4-week requirement for mental health training.^11^ As such, this is an important transitional time to bolster education on substance use screening, given the frequent co-occurrence of substance use with mental health issues among adolescents.^12^ The brief intervention component of SBIRT commonly incorporates key components of motivational interviewing (MI), such as increasing insight regarding substance use and motivation toward behavioral change.^13^ MI was selected as the primary counseling framework for our intervention because it is an evidence-based, patient-centered approach commonly used in substance use counseling. Prior systematic reviews have demonstrated that MI-based interventions are associated with reductions in substance use behaviors across a variety of populations and clinical settings, including adolescents.^14,15^ Prior work in MI training for pediatric trainees has identified that many have limited exposure to MI, typically a single lecture in medical school, highlighting the need for further training within residency.^16^

Our curriculum builds on a growing body of literature demonstrating the effectiveness of MI and SBIRT training in medical education while addressing barriers to implementation, such as time and financial constraints. Prior curricula have shown that longitudinal and skills-based MI and SBIRT training can improve participant comfort with MI skills. A prior study described a multifaceted curriculum incorporating online modules, in-person workshops, faculty feedback, and self-reflection exercises which significantly improved pediatric residents’ self-reported confidence and MI skillfulness.^17^ Similarly, another study demonstrated that structured SBIRT education using role-play, mock interviews, and supervised clinical experiences increased resident confidence and use of SBIRT practices immediately after training, although gains declined over time, highlighting the need for longitudinal reinforcement of MI education.^18^

A growing number of publications also emphasize the importance of MI education. Several published curricula specifically focus on MI training for medical trainees, spanning undergraduate medical education, residency training, and interprofessional learning.^19-25^ Existing curricula address a broad range of clinical topics, including health behavior change, chronic disease management, tobacco cessation, and general communication skills; however, relatively few focus specifically on adolescent substance use. Several published curricula have incorporated MI and SBIRT training into graduate medical education, including a 4-week SBIRT curriculum for internal medicine residents and an interprofessional adolescent substance use curriculum focused on youth with co-occurring attention-deficit/hyperactivity disorder.^24,25^ Another curriculum used standardized patient encounters to teach pediatric residents MI techniques for adolescent tobacco cessation counseling through a brief, interactive workshop.^20^ Collectively, these findings support the educational value of interactive MI training, but many rely on substantial faculty involvement, repeated observation, individualized feedback, standardized patient resources, or multiple sessions, which require protected curricular space and funding.

In contrast, our curriculum was intentionally designed as a brief, adaptable 90-minute intervention for pediatric residents that can be integrated into existing behavioral health curricula with minimal additional resources. This session is meant to be feasible for residency programs adapting to new ACGME mental health training requirements with limited curricular time. Rather than serving as a comprehensive MI curriculum, it is intended to provide additional exposure to MI while strengthening residents’ MI knowledge and self-reported comfort.^26^ By combining a concise interactive didactic with structured peer role-play and facilitated reflection, our approach emphasizes practical MI skill application. The use of role-play was designed for experiential teaching rather than formal assessment, and its use is supported by prior work as a core component of MI education.^27,28^ The use of semistructured debriefing allows for a psychologically safe learning environment with the opportunity for reflection and feedback from peers.

## Methods

This single-center educational intervention was conducted within the pediatric residency program at the Children's Hospital of Philadelphia during the 2024-2025 academic year. The residency program included 55 postgraduate year 2 (PGY-2) pediatric residents. Among these residents, 6 were medicine-pediatrics residents, 2 were pediatrics-genetics residents, 7 were pediatrics–child neurology residents, 2 participated in the global health track, and 4 were members of the Leadership in Equity, Advocacy, and Policy track. All residents participated in a 6 + 2 (“X + Y”) scheduling model in which they spent 6 weeks on inpatient blocks (“X block”) followed by 2 weeks on their ambulatory block (“Y block”); thus, residents were staggered on their Y blocks into separate Y block cohorts rather than rotating through ambulatory blocks simultaneously. During their ambulatory blocks, in addition to providing clinical care during half-day sessions at 1 of 3 primary care sites in Philadelphia, they also participated in protected didactic activities, including 3 academic half-days (AHDs) per postgraduate year. Some AHDs are part of a larger behavioral health thread, which made them a good fit for these MI case-based sessions. The MI workshop was integrated into the PGY-2 ambulatory AHD curriculum. All PGY-2 residents during the 2024-2025 academic year were assigned to a single scheduled workshop session during their Y block; however, attendance varied due to scheduling conflicts, vacation, illness, and other clinical responsibilities. This 90-minute session was the only activity for the assigned AHD. Three separate workshop sessions were delivered to unique cohorts of 10-12 PGY-2 residents between May and June 2025. There were 1-2 faculty facilitators at each session. This project was deemed exempt by the Children's Hospital of Philadelphia Institutional Review Board (IRB 25-023285). Participants provided informed consent before participation and received a $15 gift card for survey completion.

### Curriculum Overview

The material was derived from online MI training offered through the American Society of Addiction Medicine (ASAM) and the authors’ expertise in addiction medicine education.^28^ The 1.5-hour workshop included a presession survey and a 20-minute didactic session covering MI fundamentals, followed by two 30-minute structured role-play cases with facilitated debriefs, a final group discussion, and a postsurvey. Sessions were led by the curriculum developers with primary care attendings observing in preparation for future facilitation during subsequent academic years. All sessions were conducted in a conference room equipped to display presentation slides on a projector, as well as space for small-group activities. Educational materials included annotated presentation slides, a facilitator guide, role-play cases, an observer checklist, and supplementary handouts (Appendices A-H). ASAM's video-based modules, which informed the development of the didactics slides, require an additional fee for access. With permission, we reproduced the handouts used in the ASAM modules and made them freely available in the appendices to facilitate dissemination and use in residency programs.^29^ The presentation slides, facilitator guides, role-play cases, and observer checklists were developed by the authors and constitute original content. These materials incorporate frameworks and tools for MI within addiction medicine that are consistent with those presented in ASAM's educational materials.

### Workshop: Didactics and Role-Play Cases

The 20-minute didactic covered MI fundamentals, including open-ended questioning, reflective listening, readiness and confidence rulers, and strategies to identify and respond to “change talk,” “sustain talk,” and “discord.” Based on the ASAM MI framework, we incorporated the concepts of identifying different patient responses to MI by categorizing them as “change talk” (statements reflecting openness to behavior change) vs “sustain talk” (language or behavior favoring continuation of behavior) or “discord” (conflict or interpersonal tension within the clinical encounter).^29^ The session also reviewed *Diagnostic and Statistical Manual of Mental Disorders, Fifth Edition* (DSM-5) criteria for SUDs.^30^

Most of the session focused on experiential learning through 2 structured small-group role-play cases: the first was inpatient cannabis use (see Appendix C) and the second was outpatient vaping (see Appendix D). Residents were divided into groups of 3 and rotated through the roles of provider, patient, and observer. Each case was conducted twice. During the initial encounter, the patient role incorporated “change talk,” whereas the repeated encounter using the same clinical scenario emphasized “sustain talk” or “discord,” allowing residents to practice MI skills with varying communication dynamics. Case materials for the residents included example patient statements for both “change talk” and “sustain talk/discord” scenarios and ASAM's educational handouts with examples of strategies to reference as the provider (see Appendices C-F). For case 1 on inpatient cannabis use, an inpatient SHADESS (adolescent social history framework: Strengths, Home, Activities, Drug, Emotions, Sexuality, Suicidality/Safety) assessment was provided to participants as a resource to facilitate conversations as they might do in the inpatient setting (see Appendix C). For case 2, on outpatient vaping, a completed Adolescent Health Questionnaire, derived from our institution's primary care clinic screener, was provided to participants as a resource to facilitate conversation and to simulate a typical outpatient encounter (see Appendix D). All 3 participants were provided with the observer checklist for reference, but only the observing resident filled out the checklist to identify MI techniques used during encounters (see Appendix G). Checklists were used to facilitate peer feedback and discussion during the debriefing sessions; checklist data were not collected for formal analysis.

Immediately following the completion of both versions of each case, facilitators used the annotated slides and facilitator guide to lead a 10-minute large-group debrief modeled on the Promoting Excellence and Reflective Learning in Simulation (PEARLS) framework for simulation debriefing (see Appendices B and H).^31^ Debriefing emphasized reflection, psychologically safe feedback, and application of MI communication strategies.

### Workshop: Wrap-Up

The final 10-minute wrap-up reviewed common barriers to MI in clinical practice (eg, time constraints, patient readiness, clinician confidence), state confidentiality rules for adolescent substance use care, and key referral resources. Slides and facilitator notes guided the discussion of epidemiologic data and disparities in adolescent substance use to contextualize MI in clinical practice.

### Assessment and Evaluation

Participants completed pre- and immediate postintervention surveys developed by the authors to assess knowledge, attitudes, and self-reported behaviors related to MI and substance use counseling; they were not previously validated (Appendices I and J). Survey items were developed to align with the learning objectives and key MI competencies emphasized in the curriculum.

The knowledge questions were derived directly from key concepts taught in the didactic session and consisted of 5 questions, including identification of MI strategies and differentiating experimental use from a use disorder. Attitude items were designed to assess the perceived relevance of counseling using 3 Likert-style items. The behaviors section was designed to assess self-reported comfort with and use of MI skills, including counseling and referral to treatment, using 4 Likert-scale items. The postsurvey repeated these items with the addition of an open-ended section for feedback on the workshop (see Appendix J). Items were reviewed by faculty with expertise in addiction medicine and medical education to ensure content alignment.

To compare preintervention to postintervention scores, we used the following process. First, we evaluated the normality of the data with a Shapiro-Wilk test. Subsequently, parametric data were compared using a paired *t* test. Because the responses to the survey item regarding comfort with counseling was not normally distributed, nonparametric analysis with the Wilcoxon matched-pairs signed-rank test was utilized. Statistical analysis was conducted using Stata BE edition 19.0.

## Results

A total of 24 pediatric residents completed the initial pretest survey. Seventeen of the original respondents completed the immediate posttest survey after the initial intervention. Analysis of initial attitudes and experiences included all 24 initial respondents, and analysis of posttest attitudes and comparisons included only the 17 who completed both surveys.

Participants reported a variety of prior experiences with MI. Of the modalities mentioned, lecture-based learning was the most common, with 92% of trainees reporting experience with it. Standardized patient encounters were also common (79%), along with case-based learning (75%). None of the trainees surveyed reported having no experience with MI. At baseline, all surveyed participants strongly agreed or agreed that, as a pediatrician, it is their responsibility to address their patients’ substance use, and all agreed or strongly agreed that it is important that they receive training on caring for patients with substance use (*n* = 24). Participants’ self-reported comfort with using MI increased from pre- to postintervention (mean paired difference = +0.76, 95% CI: 0.25-1.28; *P* = .005). Comfort with counseling increased (mean difference = +0.71, 95% CI: 0.30-1.11; *P* = .0.003), and comfort with referring to treatment increased (mean difference = +1.00, 95% CI: 0.45-1.54; *P* = .0013). Participants also completed 5 questions to assess their knowledge of MI for SUD before and after the intervention. Knowledge scores, as defined as the number of correct questions out of 5, increased from a mean of 4.11 to 4.60 postintervention (mean paired difference = +0.47, 95% CI: 0.02-0.92; *P* = .041).

## Discussion

After this brief, interactive role-play session, participants demonstrated higher postintervention knowledge scores and reported greater comfort using MI strategies and referral to treatment for adolescent substance use. This curriculum contributes to the growing literature on pediatric MI and SBIRT education by providing an interactive, skills-based workshop focused on adolescent substance use and SUD care. The inclusion of an inpatient case broadens the application of MI techniques beyond the outpatient setting, reflecting the diverse clinical settings in which trainees practice.^27^

Overall, the findings support the feasibility of integrating targeted training into pediatric residency curricula and suggest potential benefit in improving residents’ self-reported comfort with substance use counseling, although effectiveness in improving MI proficiency was not directly assessed. Baseline exposure to MI was high among participants, with all trainees reporting prior MI experience through multiple educational modalities. Given this prior exposure, this intervention may have functioned as skills-based reinforcement rather than foundational instruction. The results should be interpreted cautiously given the small sample size, participant attrition, and wide confidence intervals for some outcomes. In addition, reliance on immediate postintervention, self-reported measures limits conclusions regarding durability of effects and potential impact on clinical practice.

Several limitations should be considered. This curriculum was implemented at a single pediatric residency program with a small sample size, which may limit generalizability. The pretest behavior items were limited to discrete counts of self-reported clinical actions, which provides a crude estimate of the frequency of MI use in clinical encounters. As such, these items should be interpreted cautiously, as they may not fully reflect actual clinical behavior or allow for meaningful comparison across participants. Outcomes were based primarily on immediate postintervention self-report measures, making results susceptible to response and social desirability bias. The use of participant identifiers may have further reduced anonymity. Furthermore, the survey instrument was developed for this project to align with the objectives and was not formally validated. The knowledge assessment included a small number of items and was designed to evaluate recognition and application of MI concepts rather than comprehensive factual knowledge of SUDs. In addition, MI skills were not directly assessed through observed encounters or standardized patient evaluations; therefore, improvements in knowledge and comfort should not be interpreted as evidence of clinical proficiency. The intervention also required trained facilitators and protected educational time, which may affect scalability in settings with fewer available faculty resources. The use of a financial incentive for survey completion may have influenced response behavior and participation. Attrition further limited paired analysis, as only 70.8% of participants had completed both pre- and postintervention assessments, limiting interpretation of potential nonresponse bias.

Overall, this intervention has the potential to be expanded to other training programs, particularly at a time when the ACGME recognizes behavioral health as an issue of growing importance within pediatrics, and to assist pediatric practitioners in addressing adolescent substance use.^2^ Based on postsession reflections from both participants and facilitators, several modifications are planned for future iterations. These include making the didactic section more interactive (eg, live polls or think-pair-share activities), assigning specific “observer tasks” during role-play, and restructuring the role-play into a continuous encounter to enhance realism and flow. Additionally, future sessions should be developed to expand focus on how to effectively include and support family members using MI-consistent strategies, such as seeking adolescent consent to involve caregivers and practicing joint goal setting. Further work should explore the applicability of this intervention in other pediatric training programs as well as repeat assessments of participants over time, ideally through observed clinical encounters. Additionally, future work should evaluate whether this intervention impacts patient-level outcomes, such as rates of screening, counseling, and referrals to substance use or other mental health services.

## Appendices


MI Didactic Slides.pptxMI Didactic Slides Annotated.pdfCase 1 - Cannabis HPI and SHADESS.docxCase 2 - Vaping HPI and AHQ.docxASAM Handout - Responding to Sustain Talk.pdfASAM Handout - Evoking Change Talk.pdfCase 1 and Case 2 Observer Checklist.docxFacilitator Guide.docxMI Pretest.docxMI Posttest.docx
*All appendices are peer reviewed as integral parts of the Original Publication.*
