# Supplementary material for: Motivational Interviewing for Pediatric Residents: A Case-Based Workshop on Adolescent Substance Use
Source: MedEdPORTAL. 2026 Oct 22;22:11641. doi: 10.15766/mep_2374-8265.11641 (PMC13687990; doi:10.15766/mep_2374-8265.11641)
Supplement: Supplementary file 1 [file mep_2374-8265.11641-s001.zip › B. MI Didactic Slides Annotated.pdf]

Using the Annotated Slides:
The following document includes detailed notes for each slide to help facilitate the “Case-Based Workshop on Adolescent Substance Use” as a primary care educator/general pediatrician. This slide deck should be reviewed prior to each workshop so that facilitators can refresh their understanding of the workshop’s objectives and content.

The Facilitator Guide (Appendix F) should be reviewed in conjunction with this guide. Many facilitators choose to have these slides available in “Presenter View” during the session to access annotations while the slides are being projected.

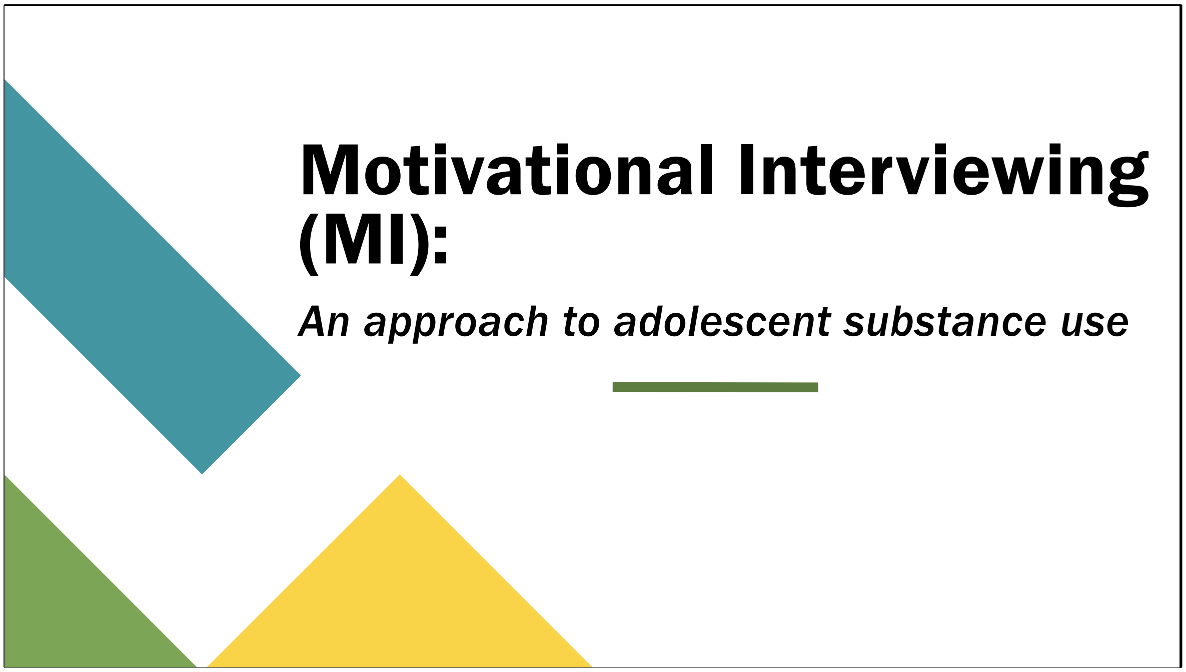

Agenda

- Brief introduction to MI (20 minutes)
- Two cases and discussion (60 minutes)
- Wrap up and debrief (10 minutes)

"We think of MI-consistent styles as dancing instead of wrestling. One moves ***with rather than against*** the person. We are not trying to overpower our patients until they surrender to change. Instead, we come alongside them and skillfully guide the conversation towards change."

- Dr. Brian Hurley, MD, MBA, DFASAM, FAPA

Objective

By the end of this workshop, learners should be able to:

- **Recognize at least 3 components** of motivational interviewing (MI) as they relate to management of substance use in adolescents
- **Apply techniques** of motivational interviewing to brief intervention in **two example clinical scenarios**
- **Distinguish a substance use disorder from experimental or problematic substance use** according to the DSM V Criteria for Substance Use Disorders (SUD)
- **Identify community resources** for referral to treatment and **discuss ways to involve caregivers** in the care plan

Pretest Survey

- Please follow the QR link to the gift card and pre-test survey
- For those who fill out the 3 surveys (2 today and 1 in 6 months), we will be providing a $15 gift card to Starbucks via the email provided.
- The post-test survey will be emailed to you to complete AFTER the session

A QR link to the pretest survey was originally placed here (Appendix J).

As part of our evaluation for this session, we will have a 2 brief surveys today and 1 in 6 months. Those who complete all 3 surveys will received an emailed $15 Starbucks gift card. Please follow the QR link to the gift card survey and pretest survey. A post test survey will immediately be emailed to you but please wait until the end of the session to complete it!

Introduction to MI

Let's talk about ambivalence

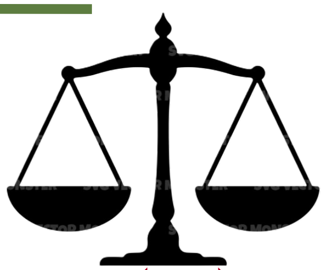

Resistance ⟷ **Change**

Image by Creazilla, retrieved from: https://creativecommons.org/publicdomain/zero/1.0/ on 1/17/2026. Image is in the public domain.

- When being pulled in multiple directions, they sometimes get *stuck.*
- Ambivalence is **normal**. It should be explored, not confronted.
- MI helps us challenge our assumptions about someone being "unmotivated"

- Ambivalence should be an expected part of MI
- It's when somebody is being pulled strongly in two different directions, and they get stuck in the middle.
- Ambivalence is normal, and it should be explored through MI.
- We can shift our thinking from, "why isn't the person motivated?" to the assumption that everyone is motivated for something and we need to help figure out "for what is the person motivated?" Ultimately, it's about figuring out what does this person want.

Engaging the patient

- Classic medical model style
- Following style
- Guiding style

- In motivational interviewing, we can think about a range of different styles.
  - The classic medical model style, is directive
  - On the other end is the following style, where we go along with the patient's thought process without offering direction or help.
  - In motivational interviewing, we tend to use more of a guiding style. To offer encouragement and help find one's own motivation to be successful at meeting your goal and getting to your destination. Like guiding up a hill.
    - Pediatrics is unique in that it may involve some directive/classic style

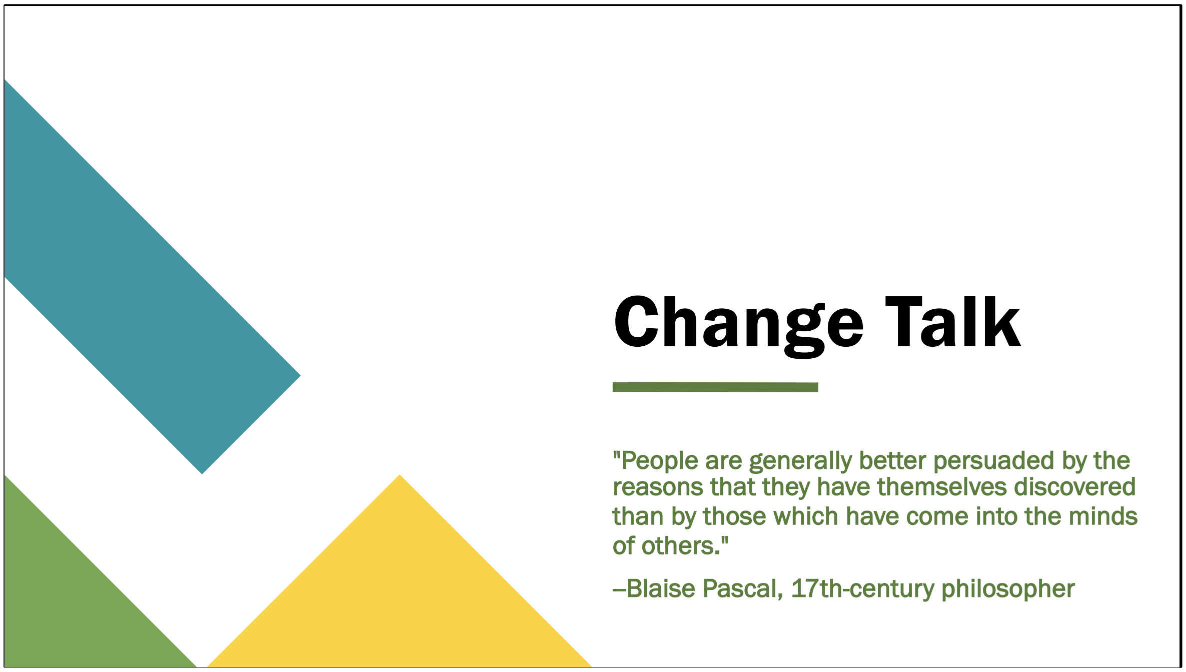

Now we can delve into the practice of MI and strategies to elicit change talk. This quote encompasses the theory of MI: "People are generally better persuaded by the reasons that *they have themselves discovered* than by those which have come into the minds of others."

Change Talk: DARN CATS

Preparatory Change Talk

DARN stands for **desire, ability, reasons**, and **need**

- DESIRE: "I want to change..." (e.g., I want, like, wish)
- ABILITY: "I can change..." (e.g., I can, could)
- REASONS: "I have good reasons to change..." (e.g., if...then...)
- NEED: "I will/have to/got to change..." (e.g., I need to, have to, got to; importance)

Mobilizing Change Talk

CATS stands for **commitment, activation,** and **taking steps**

- COMMITMENT: "I've decided to..." (i.e., intention, decision, readiness)
- ACTIVATION: "I made the call." "I made the appointment." (i.e., read, prepared, will)
- TAKING STEPS: "I'm going to meetings." (i.e., recent specific actions toward change)

- First, we will define change talk. Change talk is patient speech that favors movement in the direction of positive change. The acronym for change talk is **DARN CATS**
- DARN is the preparatory change talk, the kind of pre-contemplation into contemplation and preparation. And CATS is the preparation, action, and maintenance part of change or mobilizing change talk

Strategies: Overview

- OARS
  - Open-Ended questions, Affirmations, Reflections, Summary
- Readiness/confidence ruler and decisional balance
- Strategies to address sustain talk and discord

- In order to evoke change talk, we will review a few strategies that can be used
- We will review a few key micro-skills that keep a motivational-interviewing conversation flowing
- We will ten review how to recognize sustain talk and how to address that

OARS

- Open-ended questions
  - **Decisional balance**- advantages/disadvantages of change/staying the same
- Affirmations
- Reflections
  - Simple
  - Complex: Amplified, Double-sided
- Summary

- The OARS mnemonic is the cornerstone of MI strategies

  Open-ended questions allows the patient to elaborate/share their story, moves the conversation forward, evokes change talk
  - It explores Decisional balance: Discussing the **costs and benefits of the choices they can make**
  - You can use **decisional balance**, the **advantages of change**, the **disadvantages of change**, the **advantages of staying the same, the disadvantages staying the same**. Use the ruler technique, which we will review in a moment, as a jumping off point for these questions

- Affirmations: Affirmations are different that praise. An affirmation is highlighting the character trait or the behavior that you're trying to reinforce. Contrary to what we may be used to doing, an affirmation starts with a "you" statement, whereas praise is more about "I" statements. Ex. "You made a choice to go to group counseling, and you did it." Instead of "I'm so proud of you"

- Reflections: Reflections are statements where we repeat back to somebody what they've told us. And they come in two types which we will review a bit more in the

coming slides.

- There are simple reflections, where we just repeat back what someone told us, and we stay close to the speaker's words. If somebody said, "I'm tired of talking about this." You might say "You're tired of talking about this."
- Complex: Amplified, double-sided which we'll address in the next few slides

- Summaries are like mega-reflections. They provide a collection of thoughts, ideas, and/or feelings from present or past conversations with a patient. The goal is to link information, summarize ambivalence, or transition to another topic. Ex. "You had a very stressful day, and it triggered your urges, which led to you to buy a vape pod. You smoked less than you would have in the past and then discarded the remainders. Your health is important to you, and you want to continue to work on your vaping cessation."

Evocative Questions: Open-Ended Questions

- Ask open-ended questions
  - Try to evoke the DARN CATS "change talk"
    - Why is this change important to you? (Desire)
    - How will you cut back on vaping? (Ability)
    - What is motivating you to explore this change? (Reasons) and how important is it to you to succeed? (Need)
    - What will you do next? (Commitment)
  - Ask for examples of how their use has impacted themselves/others

- Here are some more examples of **open-ended questions** to evoke DARN CATS statements

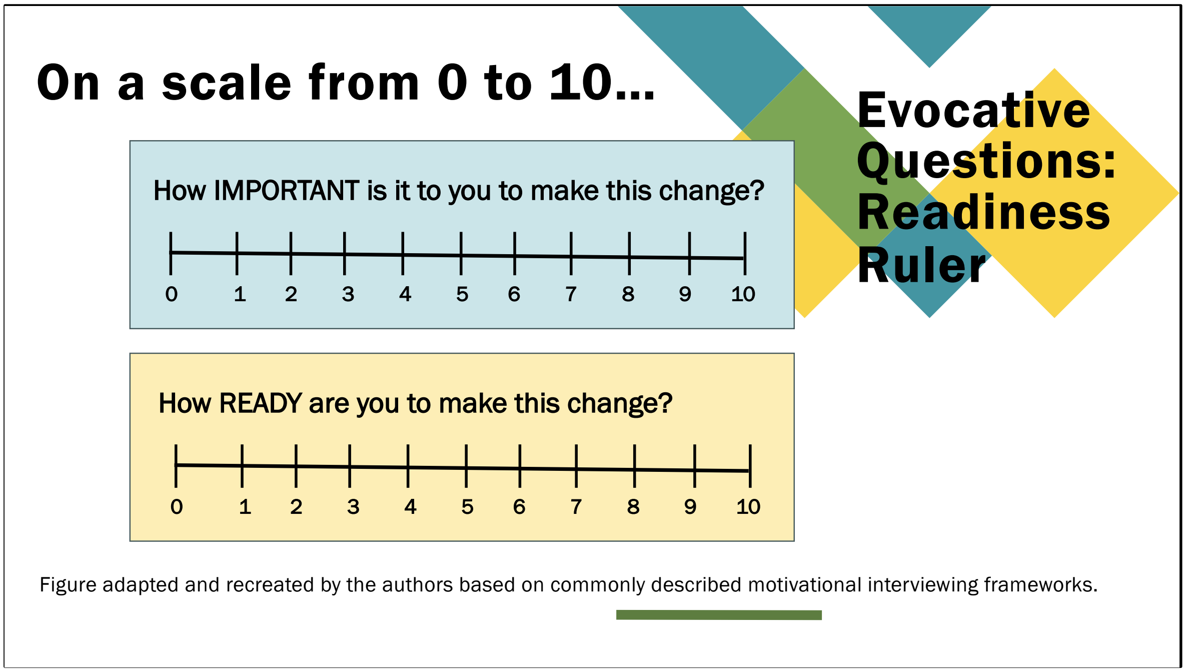

Figure adapted and recreated by the authors based on commonly described motivational interviewing frameworks.

You can use the **readiness ruler**. The readiness ruler has two parts on a scale of zero to 10? How important is it for you to change x behavior?

And then on the **confidence ruler**, or on a scale of zero to 10? How confident are you could change x.

With either, when somebody gives you a number, say they're 5 out of 10 ready, they're in that perfectly ambivalent spot into how important it is to them. You say, "Well, why is it a 5 and not a 2?" Ideally this allows them to give you information about why the change is important to them.

When they give you that five out of ten, then you can say "what would it take for you to go from a five to a seven," to evoke their ideas, their values, their intentions for what they could do?

Two Elements of Resistance: Sustain Talk and Discord

Sustain Talk

- The other side of a patient's ambivalence that argues against change or argues for preserving the status quo

Discord

- Can occur in the context of ambivalence, but it points to an interpersonal problem rather than reluctance to entertain change.
- May involve arguing, interrupting, negating, or ignoring the clinician

- When we talk to patients, we are listening for change talk. However, sometimes we get irrelevant talk, or we'll get sustain talk.
  - Sustain talk is the opposite of change talk.

- It is ok to acknowledge their sustain talk but not dwell on it or reinforce is that so that the conversation can pivot towards change talk

- They may or may not even want to have a conversation. You'll recognize discord when you hear patients say things like "It's not my fault." "It's not that bad." "You don't understand." Or if a patient just starts saying yes to everything. "Yeah." "Sure." "Yep." Those are all cues that we've lost engagement.

Strategies to Address Sustain Talk

- Simple Reflection
- Amplified Reflection
- Double-sided Reflection
- Reframing
- Emphasize Autonomy

- Simple Reflection: A simple reflection is a repeat or rephrasing of what they've said. This helps the patient understand that we hear what they are saying.
  - Patient says "I will never quit vaping." Physician responds, "You don't see yourself stopping vaping."

- Amplified Reflection: Exaggerates or increases the magnitude of what the patient has said. This is meant to encourage the patient to correct or argue against what was said in order to elicit change talk.
  - For example, Patient says “I'm here because my mom made me come here.” Physician: “That's the only reason why you're here.”

- Double sided Reflection helps reflect both sides of ambivalence
  - Patient says: “I don’t like what vaping does to my health, but it really reduces my stress.” and Physician says: “On the one hand, vaping helps calm you down, but on the other hand you’re concerned about how it affects your health."

- Reframing: validate the person's statement while offering a different perspective
  - For example, the patient says: "I’ve tried to stop before, but I only made it

three weeks." And the Physician says: "You stopped drinking for three weeks; you did that on your own."

- Emphasizing autonomy is a strategy where you emphasize to the patient that it is ultimately their decision whether they will make a change or not.

Substance Use Spectrum

STAGE	DESCRIPTION
Abstinence	Never used substances
Experimentation	The first few time of use usually driven by curiosity how the drug feels
Limited use	Using with others in a low-risk situation usually limited to weekends
Problematic use	Using in high-risk or inappropriate situations (driving, at school); used associated with consequences (arrest, suspension)
Substance use disorder	Drug use with recurrent consequences that interferes with functioning – see DSM 5 criteria

Consider breaking confidentiality

Chart recreated by the authors based on commonly defined stages of substance use.

Before we start the cases to talk about strategies, we should briefly discuss the DSM V criteria for substance use with and without disorder. It is important to get a sense of the stage of substance use when counseling patients to ultimately help decide on referral to treatment vs in office counseling.

Experimental use is occasional, curiosity-driven substance trying without significant consequences. **Problematic use** causes harm or disruption but doesn't meet full disorder criteria. **Disordered use** (substance use disorder) involves compulsive use with significant impairment and meets diagnostic criteria requiring treatment.

Diagnosis of Substance Use Disorder

1. Taking the substance in larger amounts or for longer than intended.
2. Unsuccessful attempts tocut down or stop using the substance despite trying.
3. Spending a lot of time getting, using, or recovering from use of the substance.
4. Cravings and urges to use the substance.
5. Continued use despite recurrent problems at work, home, or school because of substance use.
6. Continued use, even when it causes problems social and interpersonal problems.
7. Giving up or reducing important social, occupational, or recreational activities because of substance use.
8. Recurrent substance use, even when it puts you in danger.
9. Continuing to use, despite knowledge of a physical or psychological problem that could have been caused by or made worse by the substance.
10. Needing more of the substance to get the effect you want (tolerance).
11. Development of withdrawal symptoms, which can be relieved by taking more of the substance.

Diagnostic concepts informed by DSM-5-TR criteria (American Psychiatric Association, 2022).

- The DSM-5 criteria for substance use disorders (SUDs) include 11 criteria that fall into four categories: impaired control, physical dependence, social problems, and risky use
- Here is an example from Peds in Review article about substance use using cannabis use disorder - on the next slide we will simplify how to identify a use DISORDER

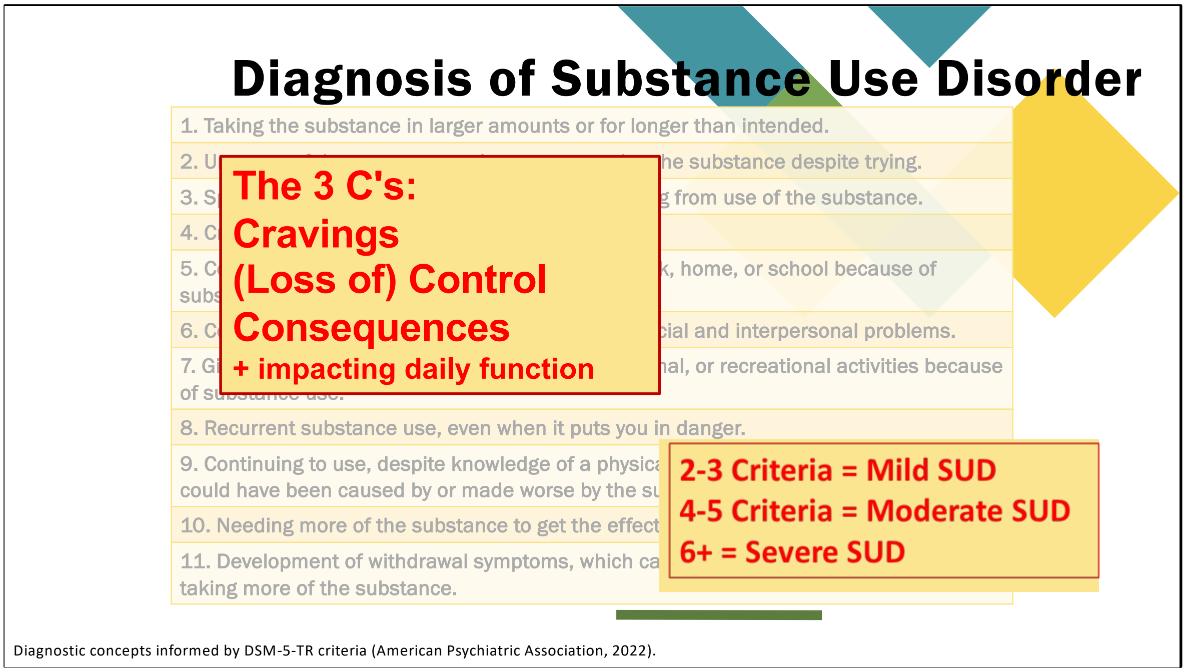

- The DSM-5 criteria for substance use disorders (SUDs) include 11 criteria that fall into four categories: impaired control, physical dependence, social problems, and risky use
- This can be an easier way to quickly identify a use disorder, you can always revisit the full criteria if concern arises

1. **Craving (or Compulsion to Use)**
   - A strong desire or urge to use the substance.
   - Cravings can be physical or psychological and are often triggered by stress, environment, or specific cues.

2. Control (Loss of Control)
   - The individual uses more of the substance than intended or for a longer period than planned.
   - Repeated unsuccessful efforts to cut down or control use.
   - Substance use may feel compulsive or out of the person's control.

3. **Consequences (Continued Use Despite Negative Consequences)**
   - The person continues to use the substance despite experiencing harmful effects, such as:
   - Health issues

- Strained relationships
- Legal problems
- Poor performance at work or school

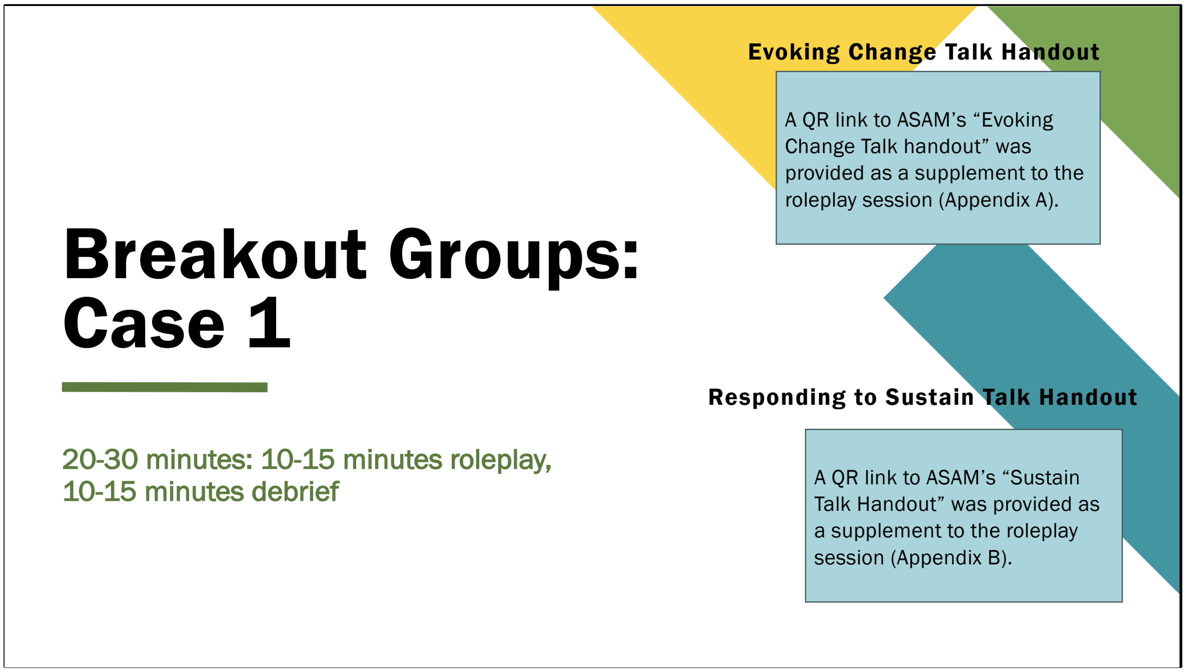

Residents will break up into groups of 3-4 to roleplay and observe 2 cases. One resident will be the patient, one the provider, and one the observer. There are 15-20 minutes for each case followed by 10 minutes to debrief. Please keep track of time with each transition.

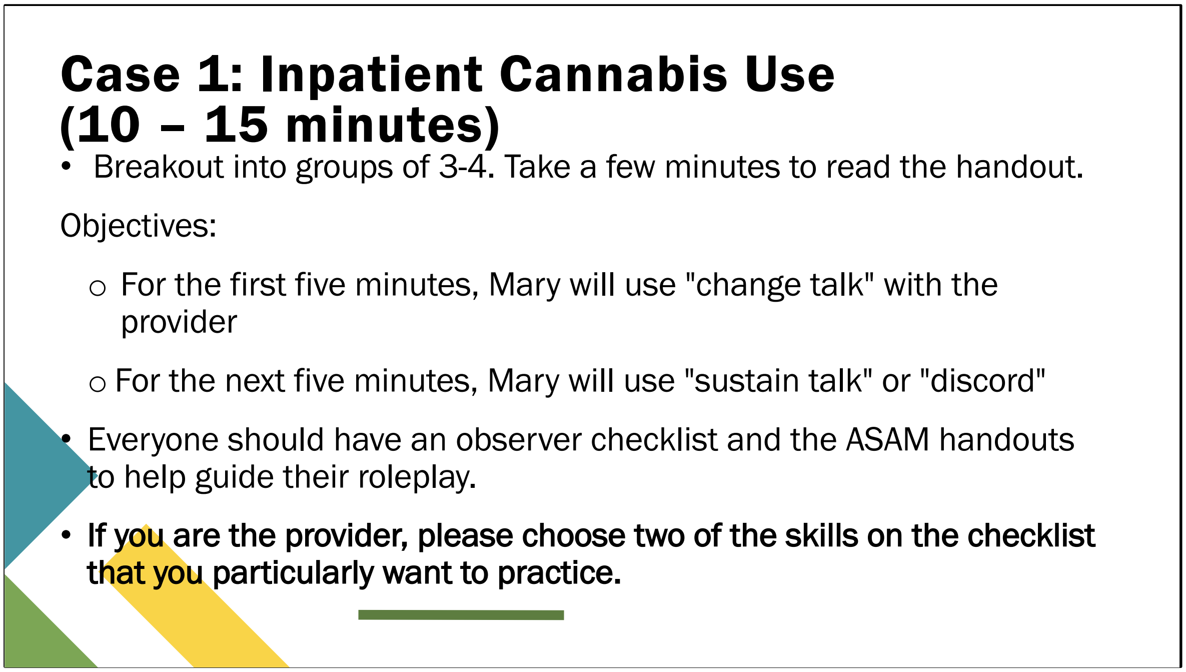

Case 1 is about inpatient cannabis use. Residents will receive the case, a SHADESS handout, and an observer checklist. QR codes in the powerpoint will link to ASAM MI interviewing tips. Have residents self-assign roles. The provider should identify 2 skills from the checklist that they would like to employ during the roleplay session. The case will run twice for 5 minutes each time and trainees should switch roles with the second iteration of the encounter. During the first session, Mary will embrace “change talk,” which will make the interaction relatively simple. After 5 minutes, have the residents switch roles and this time, Mary will embrace “sustain talk” and/or “discord” with the provider.

Debrief: Case 1 (10 – 15 minutes)

:

Let's reconvene as a larger group to talk about Case 1.

- How did that feel? How did the "change talk" scenario compare to the "sustain talk/discord" scenario?
- Let's hear from the observers, which strategies did your group use? Which ones worked well?
- Did anyone use the confidence or readiness ruler? How did it go?

Breakout Groups: Case 2

20-30 minutes: 10-15 minutes roleplay, 10-15 minutes debrief

Evoking Change Talk Handout

A QR link to ASAM's "Evoking Change Talk handout" was provided as a supplement to the roleplay session (Appendix A).

Responding to Sustain Talk Handout

A QR link to ASAM's "Sustain Talk Handout" was provided as a supplement to the roleplay session (Appendix B).

Case 2: Outpatient Vaping (10 – 15 minutes)

- Breakout into groups of 3-4. Take a few minutes to read the handout.

Objectives:

- o For the first five minutes, Nico will use "change talk" with the provider
- o For the next five minutes, Nico will use "sustain talk" or "discord"

- Everyone should have an observer checklist and the ASAM handouts to help guide their roleplay.
- **If you are the provider, please choose two of the skills on the checklist that you particularly want to practice.**

Case 2 is about outpatient vaping. Have residents self-assign roles: one observer, one patient, one as the provider/interviewer. Residents will receive the case, an adolescent health questionnaire, and an observer checklist. The provider/interviewer should identify 2 skills from the checklist that they would like to employ during the roleplay session. QR codes in the powerpoint will link to ASAM MI interviewing tips. The case will run twice for 5 minutes each time; have learners switch roles with the second iteration of the case. During the first session, Nico will embrace “change talk,” which will make the interaction relatively simple. After 5 minutes, have the residents switch roles and this time, Nico will embrace “sustain talk” and/or “discord” with the provider.

Debrief: Case 2 (10 – 15 minutes)

Let's reconvene as a larger group to talk about Case 2.

- How did that feel? How did the "change talk" scenario compare to the "sustain talk/discord" scenario?
- Let's hear from the observers, which strategies did your group use? Which ones worked well?
- Did anyone use the confidence or readiness ruler? How did it go?
- How would you approach involving Nico's parents?

Wrap Up!

10 Minutes

Overcoming barriers

Common Barriers to Practicing MI

What ASAM's Survey Research Reveals about Clinicians' Concerns about Using Motivational Interviewing

- Time constraints with patients
- Difficult or unwilling patients
- Lack of confidence in motivational interviewing skills

- **Time Constraints:** Even if a conversation does not end in a plan, these conversations can build upon each other and stir up introspection. Using open questions and reflections, allows you to effectively get the information that you need to know.

- **Difficult or unwilling patient**: Reframe your thinking with this- instead of exploring where the patient is, you may be stuck on fixing it. There needs to be overlap with the patient's agenda and your agenda - focus on their priorities and ask permission before giving your input.

- **Lack of confidence in motivational interviewing skills:** Practice is important, but also embracing this framework sets the stage for appropriate use of core MI counseling skills such as open-ended questions, affirmations, reflections, and summarizing (OARS)

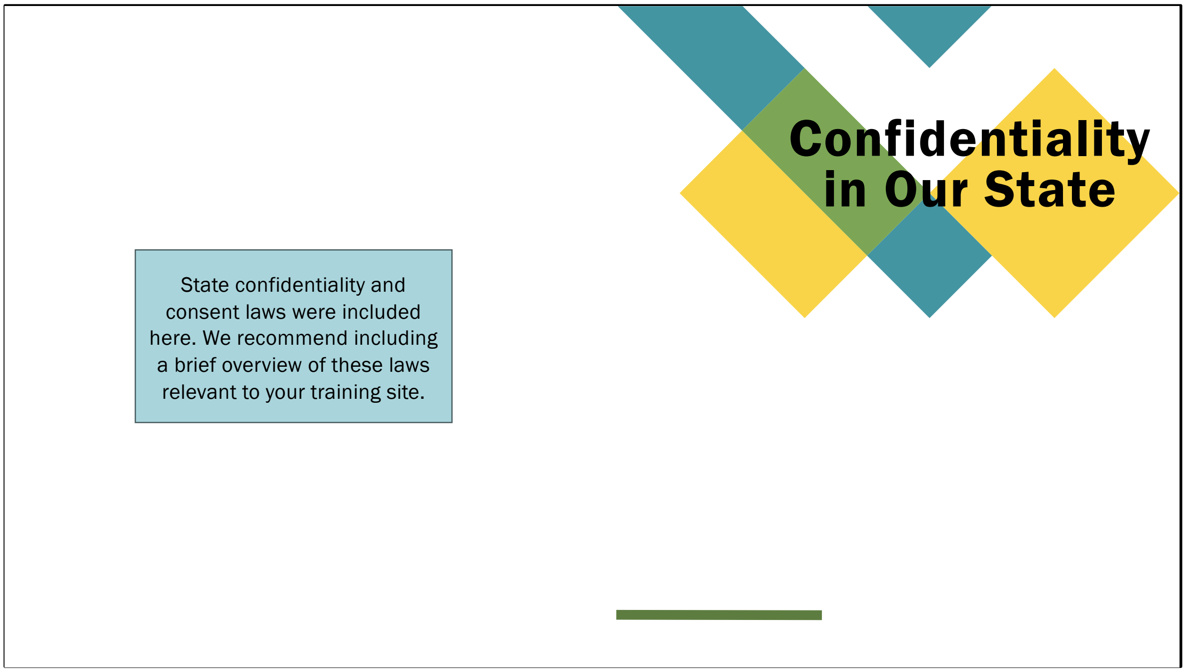

- When safe and appropriate, encourage adolescents to **disclose substance use** to caregivers.
- Minors can **consent to medical care or counseling** for drug/alcohol use without parental involvement.
- Clinicians **are not required to inform parents/guardians** unless:
  - The minor **lacks decision-making capacity**, or
  - There is a **substantial threat** to the life or physical well-being of the minor or others.

Defining "substantial threat" is a bit of a gray area but things to consider include:
(1) if anyone is using opioids, benzo's, pills or enough alcohol that withdrawal would be life-threatening due to risk of overdose or death
(2) Comorbid depression/active SI
(3) Other minors in the home with a hidden supply that could be dangerous

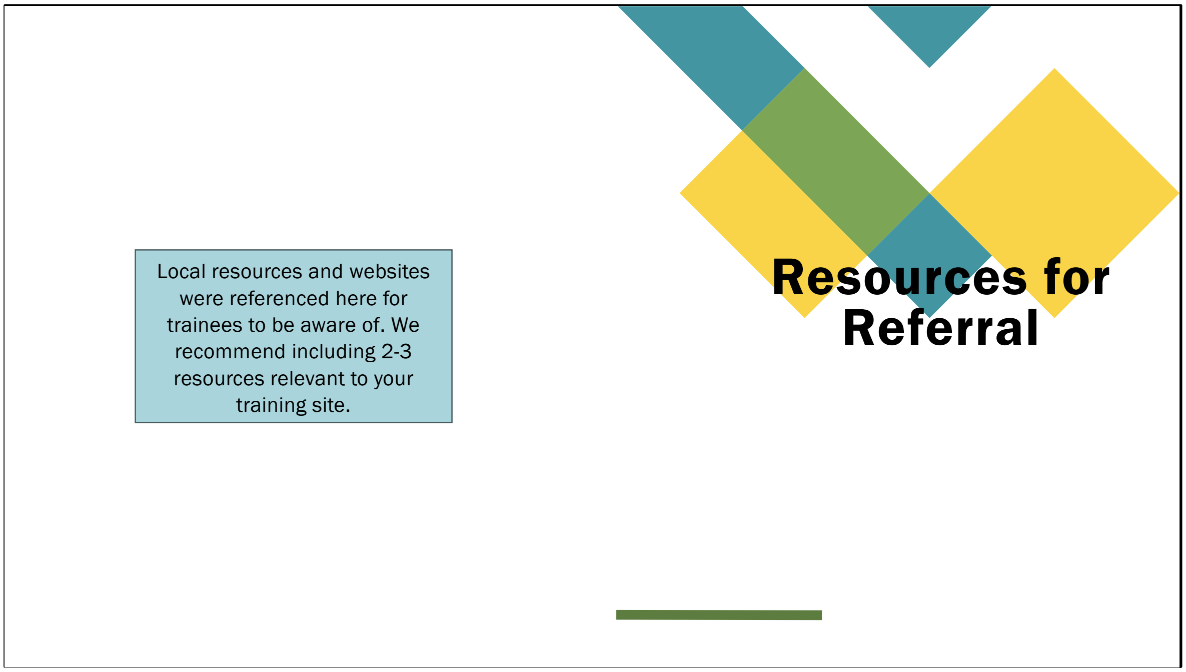

It is important to know the local agencies and orgs who treat teens for wherever you end up practicing. Here are some websites to explore.

We have created a dot phrase with these resources that we will share with you all.

- Overdose deaths among adolescents 14 to 18 years of age ***more than doubled between August 2019 and March 2020*** [4]
- Drug overdoses and poisonings are now the third-leading cause of pediatric deaths in this country [4]
- Large disparities exist in overdose deaths based on race and ethnicity. For example, the mortality for non-Hispanic American Indian or Alaska Native adolescents was 1.82 times as high as that among non-Hispanic White adolescents between 2020 and 2022 [4]

Epidemiology & Disparities

We've seen a really sharp rise in overdose deaths among adolescents—these deaths more than doubled in a short span of time. Overdoses are now the third-leading cause of death for youth in the U.S., which is alarming on its own. But what's even more troubling is that there are big disparities by race and ethnicity, with Native American and Alaska Native teens being especially affected.

- Rates of SUD are similar in white vs. racially minoritized populations
- Rates of SUD are higher in gender and sexual minority youth compared to cisgender and heterosexual counterparts[7]
- **Rates of treatment are much lower in minoritized populations[1,9]**

Epidemiology & Disparities

Even though substance use disorder rates are pretty similar between white youth and youth from racially minoritized groups, treatment access really isn’t equal. Gender and sexual minority youth actually have higher rates of SUD, but across the board, it’s the minoritized populations who are much less likely to get the care they need.

Post-test Survey

The survey in Appendix I was automatically emailed to participants 2 hours after the pretest as a linked survey from the initial QR code.

Thank you for your participation!

References

1. Alegria, M., Carson, N. J., Goncalves, M., & Keefe, K. (2011). Disparities in treatment for substance use disorders and co-occurring disorders for ethnic/racial minority youth. Journal of the american academy of Child & adolescent Psychiatry, 50(1), 22-31.

2. American Psychiatric Association. (2022). *Diagnostic and statistical manual of mental disorders* (5th ed., text rev.). American Psychiatric Publishing. https://doi.org/10.1176/appi.books.9780890425787

3. Bearnot, B., Devido, J., Hurley, B., Marienfeld, C. & Ponce Martinez, C. (n.d.). ASAM Motivational Interviewing Mini Courses Online [MOOC]. ASAM. https://elearning.asam.org/products/asam-motivational-interviewing-mini-courses#tab-product_tab_overview

4. Boykan R, Gorzkowski J, Marbin J, Winickoff J. Motivational Interviewing: A High-Yield Interactive Session for Medical Trainees and Professionals to Help Tobacco Users Quit. *MedEdPORTAL*. 2019;15:10831. https://doi.org/10.15766/mep_2374-8265.10831

5. Confidentiality of Substance Use Disorder Patient Records, PA Health and Human Services Department. 42 CFR 2.14 (82 FR 6115, Jan. 18, 2017, as amended at 89 FR 12621, Feb. 16, 2024).

6. Friedman J, Godvin M, Shover CL, Gone JP, Hansen H, Schriger DL. Trends in Drug Overdose Deaths Among US Adolescents, January 2010 to June 2021. JAMA. 2022;327(14):1398–1400. doi:10.1001/jama.2022.2847

7. Friedman J, Hadland SE. The Overdose Crisis among U.S. Adolescents. N Engl J Med. 2024 Jan 11;390(2):97-100. doi: 10.1056/NEJMp2312084. Epub 2024 Jan 6. PMID: 38198189; PMCID: PMC10979414.

8. Kirstin A. M. Nackers, Patricia Kokotailo, Sharon J. L. Levy; Substance Abuse, General Principles. Pediatr Rev December 2015; 36 (12): 535–544. https://doi.org/10.1542/pir.36-12-535

9. Kaushal Dosani, Carolina Koletic, Rabea Alhosh; Cannabinoid Hyperemesis Syndrome in Pediatrics: An Emerging Problem. Pediatr Rev September 2021; 42 (9): 500–506. https://doi.org/10.1542/pir.2019-0097

10. Lu, W., Lopez-Castro, T., & Vu, T. (2023). Population-based examination of substance use disorders and treatment use among US young adults in the National Survey on drug use and health, 2011–2019. Drug and Alcohol Dependence Reports, 8, 100181.

11. Mereish EH. Substance use and misuse among sexual and gender minority youth. Curr Opin Psychol. 2019 Dec;30:123-127. doi: 10.1016/j.copsyc.2019.05.002. Epub 2019 May 18. PMID: 31202102; PMCID: PMC6859198.

12. PROS (Pediatric Research in Office Settings)/University of Michigan BMi² (Obesity Prevention) Study, "Motivational Interviewing", Implementing Mental Health Priorities in Practice

13. Sharon J.L. Levy, Janet F. Williams, COMMITTEE ON SUBSTANCE USE AND PREVENTION, Sheryl A. Ryan, Pamela K. Gonzalez, Stephen W. Patrick, Joanna Quigley, Lorena Siqueira, Vincent C. Smith, Leslie R. Walker; Substance Use Screening, Brief Intervention, and Referral to Treatment. Pediatrics July 2016; 138 (1): e20161211. 10.1542/peds.2016-1211

14. SUD Confidentiality. Department of Drug and Alcohol Programs. (2022, July 25). https://www.ddap.pa.gov/Pages/SUD-Confidentiality.aspx
