# Supplementary material for: Motivational Interviewing for Pediatric Residents: A Case-Based Workshop on Adolescent Substance Use
Source: MedEdPORTAL. 2026 Oct 22;22:11641. doi: 10.15766/mep_2374-8265.11641 (PMC13687990; doi:10.15766/mep_2374-8265.11641)
Supplement: Supplementary file 1 [file mep_2374-8265.11641-s001.zip › G. Case 1 and Case 2 Observer Checklist.docx]

**Using the Observer Checklist**
The following document is for the observer within each small group. Participants will be divided into groups of three and rotate through the roles of provider, patient, and observer. The observer should tally the number of motivational interviewing techniques they observe during the role-play on this checklist during Case 1 and Case 2 (Appendices C and E, respectively). Before starting the role-play, the provider should choose two of the skills on the checklist to practice.

*If you are the observer, please tally the number of MI skills used and jot down notes about the approach used.*

***If you are the provider, please choose two of the skills on the checklist that you particularly want to practice.***

| MI Principle (ex:...) | Used? (Y/N) | Comments |
| --- | --- | --- |
| Open-ended question  Ex. Why would you want to make this change? What are your reasons for changing/not changing? |  |  |
| Use of the Readiness Ruler  Ex. On a scale of 1-10 how important is it for you to change this behavior? What makes you say [patient’s #] rather than [lower #]? |  |  |
| Decisional Balance: Pros, cons  Ex. What are the advantages/disadvantages to stopping? What are the advantages/disadvantages to  continuing? |  |  |
| Elaboration: Asking for examples  Ex. In what ways does [behavior] impact you? |  |  |
| Simple Reflection |  |  |
| Amplified Reflection  Ex. Exaggerating the magnitude of what the patient has said |  |  |
| Double-Sided Reflection  Ex. "On one hand... and on the other hand..." |  |  |
| Reframing:  Validating patient’s statements while offering a different perspective |  |  |
| Emphasizing Autonomy:  Emphasizing to the patient that the decision to make change is ultimately theirs  Ex. “And that is your choice to make. Ultimately the decision is yours.” |  |  |
