# Supplementary material for: Motivational Interviewing for Pediatric Residents: A Case-Based Workshop on Adolescent Substance Use
Source: MedEdPORTAL. 2026 Oct 22;22:11641. doi: 10.15766/mep_2374-8265.11641 (PMC13687990; doi:10.15766/mep_2374-8265.11641)
Supplement: Supplementary file 1 [file mep_2374-8265.11641-s001.zip › H. Facilitator Guide.docx]

**Using the Facilitator Guide:**
The following document details how to prepare for and facilitate the “Case-Based Workshop on Adolescent Substance Use” as a primary care educator/general pediatrician. This Facilitator Guide should be reviewed prior to each workshop so that facilitators can refresh their understanding of the workshop’s objectives and content.

The facilitators responsibilities prior to each workshop may include printing the cases (Appendix C, E), ASAM reference sheets (Appendix A, B) and observer checklist (Appendix D) for each small group, as well as preparing a conference room equipped to display presentation slides (Appendix G) on a projector as well as space for small group activities. Additionally, this Facilitator Guide provides an outline of the workshop with recommended timeframes and questions to ask during the debrief and wrap-up. The beginning includes a script to set the stage for small group role-play. The annotated slides (Appendix H) should be reviewed in conjunction with this guide.

Agenda

Brief introduction to MI (20 minutes)

Two cases and discussion (60 minutes)

Wrap-up and debrief (10 minutes)

Learning Objectives:

By the end of this workshop, learners should be able to:

- **Recognize at least 3 components of motivational interviewing (MI)** as they relate to the management of substance use in adolescents
- **Apply techniques of motivational interviewing** to brief intervention in two example clinical scenarios
- **Distinguish a substance use disorder from experimental or problematic substance use** according to the DSM V Criteria for Substance Use Disorders (SUD)
- **Identify community resources** for referral to treatment and **discuss ways to involve caregivers** in the care plan

**Overview**

This academic half-day session is meant to be an introduction to the strategies used in motivational interviewing. The material was adapted from the American Society of Addiction Medicine’s Motivational Interviewing Mini Courses. The slides will go through basic tenets of MI, several strategies with examples, and the DSM V criteria for substance use disorders. This will take roughly 20 minutes.

Residents will then break out into small groups of 3-4 to practice these strategies with two example cases (see below). The point of this session is to practice the skills of MI rather than discuss acute medical management. Each case will be followed by a quick debrief as a larger group.

Following the cases, there will be a wrap-up for 10 minutes reviewing barriers to MI, confidentiality rules in PA, resources, and epidemiology.

**MI Introductory Slides (20 Minutes)**

Please follow the annotated slides handout to go through the slides with the residents (can also be found in the notes section in presenter mode- it is encouraged to bring your own laptop to read off the presenter mode or a printout of the slides notes).

**Cases Overview**

These cases were developed with a focus on motivational interviewing and brief intervention as communication skills for trainees. Facilitators should preface with learners that today’s discussions will be surrounding substance use, though MI skills can be used in other contexts for pediatric providers. The cases will allow for an initial introduction to MI for substance use, which can be a very complicated topic given frequent comorbid mental health issues and the compounding effects of social determinants of health. We hope to explore these complexities in future iterations of this session and contextualize these complexities during the wrap-up.

Facilitators should use the cases and prompts to challenge the residents and yourselves in traditional “abstinence-first” anticipatory guidance and try to use MI skills to guide the patient in scenarios. Remember the learning goals above, which will help the residents apply what they learn in this case to others like it in their practice.

Residents will break up into groups of 3-4 to role-play and observe 2 cases. One resident will be the patient, one the provider, and one the observer. There are 15-20 minutes for each case, followed by 10 minutes to debrief. Please keep track of time with each transition.

**Case 1 Breakout Group (10-15 minutes)**

**Using Case 1:**
Case 1 is about inpatient cannabis use. The following case handout used by the participants. Participants will be divided into groups of three and rotate through the roles of provider, patient, and observer. All three participants should review medical background and inpatient SHADESS (adolescent social history framework: Strengths, Home, Activities, Drug, Emotions, Sexuality, Suicidality/Safety) assessment of case 1 prior to starting the role-play task. The observer should tally the number of motivational interviewing techniques they observe during the role-play on the checklist Appendix D (Case 1 and Case 2 Observer Checklist). Before starting the role-play, the provider should choose two of the skills on the checklist to practice. After participants read the case, the “provider” and “patient” will engage in a 5-minute patient encounter embracing “change talk.” They can reference Appendix A for example statements (ASAM Handout – Evoking Change Talk). For the next 5 minutes, they will reassign all three roles and engage in a 5-minute patient encounter that involved “discord” and “sustain talk” and another person will be the inpatient provider. hey can reference Appendix B for example statements (ASAM Handout- Responding to Sustain Talk).

Case 1 – Cannabis Use

Mary is a 15-year-old patient admitted to General Pediatrics for uncontrolled vomiting.

Below is a summary of her case:

ED Course

She was in her usual state of health until 2 days prior to presentation, when she developed nausea and vomiting. She has been vomiting 10-15 times a day with associated sweating and diffuse, intermittent 4/10 abdominal pain. ROS is negative for fever, rash, diarrhea, or respiratory symptoms. She tried Tylenol at home without relief. Hot showers provide temporary relief. Given that her symptoms have not improved, she presented to the ED.

Her oral intake has been inadequate, and she notes decreased urine output. There have not been any known sick contacts. She endorses smoking cannabis daily. This is her third presentation for similar symptoms in the last 6 months.

In the CHOP ED, her vital signs were initially Temp: 37.4 C HR: 124, RR: 18, and BP: 100/72. SpO2 was 100% on RA. Physical exam was notable for dry mucous membranes and mild diffuse tenderness to palpation on abdominal exam without involuntary guarding or rebound.

Labs were notable for elevated BUN, mild leukocytosis, and normal lipase and liver enzymes. Abdominal radiograph and abdominal ultrasound were unremarkable. UDS was positive for cannabinoids. She was given a bolus of normal saline and 2 doses of Zofran. Given continued vomiting, she was started on continuous fluids and admitted to the floor for further management.

Floor Course:

While on the floor, after discussing a broad differential, including potential gastrointestinal, metabolic, endocrinologic, and CNS conditions that can present with vomiting and abdominal pain, it is ultimately concluded that Cannabinoid Hyperemesis Syndrome is the most likely diagnosis. She improves with time while on intravenous hydration therapy, anti-emetics, and topical capsaicin application. You complete a SHADSSS (see document).

Task:

In groups, have a conversation with Mary for ~10 minutes, practicing the skills of motivational interviewing while assessing Mary’s readiness to change her cannabis use behaviors.

For the first 5 minutes, one person will role-play as Mary while embracing “change talk” and another person will be the inpatient provider.

For the next 5 minutes, assign new roles. One person will role-play as Mary while utilizing “discord” and “sustain talk,” and another person will be the inpatient provider.

Everyone should have an observer checklist and the ASAM handouts to help guide their roleplay.

If you are the provider, please choose two of the skills on the checklist that you particularly want to practice.

The observer’s role is to tally the number of motivational interviewing techniques they observe to help facilitate the debrief.

Example statements as Mary for “Change Talk”

Preparatory talk:

- Desire: I want, I wish, I would like to, It’d be nice if
- Ability: I can, I could, I am able to
- Reasons
- Need: I need to, I have to, I must
- Commitment: I am determined to, I am prepared to, I intend to
- Action: I am ready to
- Taking Steps: I stopped, I got rid of

Example statements as Mary for “Sustain Talk” and Discord

- My smoking is not that bad.
- It really isn’t that bad for me
- It’s the only thing that makes me feel normal
- Yup, yeah, sure, okay

**Case 1: Debrief (10-15 minutes)**

After 10 minutes, reconvene the large group to debrief the case. Start off the debrief by emphasizing that this is a safe space to ask questions and work together to figure out what went well, what did not go well, and ways to improve. This is modified from the PEARLS format for debriefing stimulation ([linked here)](https://journals.lww.com/simulationinhealthcare/fulltext/2015/04000/promoting_excellence_and_reflective_learning_in.7.aspx).

Start broadly: What went well, how did it feel, what did not go so well? What would you have changed and why?

Specifically ask about different strategies:

- Were there open-ended questions? Examples:
  - Why would you want to make this change? (Desire)
  - How might you go about it to succeed? (Ability)
  - What are the three best reasons for you to do it? (Reasons)
  - How important is it for you to make this change? (Need)
  - So what do you think you’ll do? (Commitment)
- Did they use the readiness/confidence rulers? If not, was there a missed opportunity to do so? Review use of readiness ruler if needed:
  - Ask “What makes you say [patient’s#], rather than [lower #]?”
  - Ask “What would it take for you to go from a [patient’s #] to [higher #]?”
- Other strategies to discuss:
  - Decisional Balance: Pros, cons
  - Elaboration: Asking for examples
    - Look forward and back
  - Simple Reflection
  - Amplified reflection
    - Ex. “It’s a stress reliever.” “Cannabis is the only thing that calms you down”
  - Double-Sided Reflection
    - Ex. “On one hand....on the other hand”
  - Reframing
    - Ex. “I’ve never been able to find a way to stop.” “You continue to think about ways to stop smoking marijuana because this is important to you.”
  - Emphasizing autonomy

**___________________________________________________________**

**Case 2 Breakout Group (10-15 minutes)**

**Using Case 2:**
Case 2 is about outpatient vaping. The following case handout will be used by the participants. Have participants reassign the roles of provider, patient, and observer. All three participants should review medical background and Adolescent Health Questionnaire of case 2 prior to starting the role-play task. The observer should tally the number of motivational interviewing techniques they observe during the role-play on the checklist Appendix D (Case 1 and Case 2 Observer Checklist). Before starting the role-play, the provider should choose two of the skills on the checklist to practice. After participants read the case, the “provider” and “patient” will engage in a 5-minute patient encounter embracing “change talk.” They can reference Appendix A for example statements (ASAM Handout – Evoking Change Talk). For the next 5 minutes, they will reassign all three roles and engage in a 5-minute patient encounter that involved “discord” and “sustain talk” and another person will be the inpatient provider. hey can reference Appendix B for example statements (ASAM Handout- Responding to Sustain Talk).

Case 2 – Vaping

Nico is a 17-year-old patient who you are seeing in the clinic for a routine well child visit. You have met him once before for his 15-year-old visit. Both of his parents came with him today. You take a minute to review his Adolescent Health Questionnaire (AHQ) screener (see document), and you notice that he states he is vaping nearly every day, which was not the case at your last visit together. You are unsure if his parents are aware of his vaping.

Task:

In groups, have a conversation with Nico for ~10 minutes, practicing the skills of motivational interviewing while assessing Nico’s readiness to change his vaping.

For the first 5 minutes, one person will role-play as Nico, embracing “change talk,” while another person will be the primary care provider.

For the next 5 minutes, assign new roles. One person will roleplay as Nico while utilizing “discord” and “sustain talk” and another person will be the primary care provider

Everyone should have an observer checklist and the ASAM handouts to help guide their role-play.

If you are the provider, please choose two of the skills on the checklist that you particularly want to practice.

While debriefing, consider how you would involve Nico’s parents during the encounter.

Example statements as Nico for “Change Talk”

Preparatory talk:

- Desire: I want, I wish, I would like to, It’d be nice if
- Ability: I can, I could, I am able to
- Reasons
- Need: I need to, I have to, I must
- Commitment: I am determined to, I am prepared to, I intend to
- Action: I am ready to
- Taking Steps: I stopped, I got rid of

Example statements as Nico for “Sustain Talk” and Discord

- My vaping is not that bad.
- It really isn’t that bad for me
- It’s the only thing that makes me feel normal
- Yup, yeah, sure, okay

**Case 2: Debrief (10-15 minutes)**

After 10 minutes, reconvene the large group to debrief the case. Start off the debrief by emphasizing that this is a safe space to ask questions and work together to figure out what went well, what did not go well, and ways to improve. This is modified from the PEARLS format for debriefing stimulation ([linked here)](https://journals.lww.com/simulationinhealthcare/fulltext/2015/04000/promoting_excellence_and_reflective_learning_in.7.aspx).

Start broadly: What went well, how did it feel, what did not go so well? What would you have changed and why?

Specifically ask about different strategies:

- Were there open-ended questions? Examples:
  - Why would you want to make this change? (Desire)
  - How might you go about it to succeed? (Ability)
  - What are the three best reasons for you to do it? (Reasons)
  - How important is it for you to make this change? (Need)
  - So what do you think you’ll do? (Commitment)
- Did they use the readiness/confidence rulers? If not, was there a missed opportunity to do so? Review use of readiness ruler if needed:
  - Ask “What makes you say [patient’s#], rather than [lower #]?”
  - Ask “What would it take for you to go from a [patient’s #] to [higher #]?”
- Other strategies to discuss:
  - Decisional Balance: Pros, cons
  - Elaboration: Asking for examples
    - Look forward and back
  - Simple Reflection
  - Amplified reflection
    - Ex. “It’s a stress reliever.” “Cannabis is the only thing that calms you down”
  - Double-Sided Reflection
    - Ex. “On one hand....on the other hand”
  - Reframing
    - Ex. “I’ve never been able to find a way to stop.” “You continue to think about ways to stop smoking marijuana because this is important to you.”
  - Emphasizing autonomy
- Discuss how people would go about involving Nico’s parents
  - Ask permission of Nico to include his parents in the conversation - “can we open this discussion with your parents to see if they have any ideas on how we can support your goals?”
  - Utilize MI techniques with parents – why they may want Nico to change? What tools have they used in similar scenarios?
  - May give learners permission to speak with parents independently about ways to support Nico making changes – limiting unsupervised time or access to car/transportation in times when he may be under the influence, adding consequences/incentives related to use
  - Text/technology support programs for parents
  - One evidence-based program: CRAFT Community Reinforcement and Family Training
    - Teaches skills of positive communication, rewarding positive behaviors and enrichment/self-care of caregivers/parents/guardians/other caring support people
    - https://www.overdoselifeline.org/opioid-training-and-courses/craft-family-support-training/

**Wrap-Up (10 minutes)**

Following the cases, there will be a wrap-up for 10 minutes reviewing barriers to MI, confidentiality rules in our state, resources and epidemiology. Please follow the slides and their notes.
