# Supplementary material for: Motivational Interviewing for Pediatric Residents: A Case-Based Workshop on Adolescent Substance Use
Source: MedEdPORTAL. 2026 Oct 22;22:11641. doi: 10.15766/mep_2374-8265.11641 (PMC13687990; doi:10.15766/mep_2374-8265.11641)
Supplement: Supplementary file 1 [file mep_2374-8265.11641-s001.zip › J. MI Posttest.docx]

# Motivational Interviewing Posttest

Thank you for participating in this survey to help us understand how this workshop on Motivational Interviewing may impact knowledge, attitudes, and practice of pediatric trainees.

By completing this survey, you agree to be an anonymous participant in this medical education study. Participation is voluntary and responses will not be shared with supervisors.

Participant ID:
Use the first 2 letters of first name + First 2 letters of last name + MMDD of birthdate
Ex. Jane Smith born April 12 will have the ID = JaSm0412

__________________________

**The following questions will assess your knowledge regarding motivational interviewing (MI).**

1. Which of the following statements accurately represents motivational interviewing?

- Change is marked by criticism, confrontation, and analysis.
- Change is marked by authority, brainstorming, and creativity.
- Change is extrinsic and accelerated by praise and rewards.
- Change is natural, intrinsic, and accelerated by a supportive, guiding approach.

2. Which of the following patient statements reflects the “reason” component of preparatory change talk?

- “I would like to stop vaping.”
- “I think I could cut down the number of cigarettes.”
- “I was so hungover that I overslept and missed practice again.”
- “It’s important to me to stop drinking.”

3. Which is an example of responding to an amplified reflection?
   “It feels pointless to try to quit drinking again.” - The patient

- “It sounds like you're feeling uncertain about quitting smoking.”
- “So you’re saying there's absolutely no benefit to quitting alcohol at all.”
- “You’ve been thinking about cutting back but aren't sure how to start.”
- “Stopping drinking would be difficult, but you're open to options.”

4. The following exchange is an example of responding to sustain talk by using reframing: “I’ve tried quitting so many times, and every time, I’ve failed.” - The patient

- “You’ve tried to quit so many times.”
- “You’ve never stopped. Not even for a day.”
- “And it’s your choice whether you want to try again.”
- “You’re persistent and making this change is important to you.’

5. Which of the following scenarios best represents experimental use rather than a substance use disorder?

- A college student tries cannabis once at a party out of curiosity and does not use it again.
- A teenager continues to drink four drinks after school daily despite negative consequences at school and home.
- A college student intends to cut back on drinking but despite attempts is experiencing cravings and withdrawal symptoms when not using alcohol.
- A young adult repeatedly uses cocaine despite unsuccessful attempts to cut down.

-----------------------------------------

**The following questions will assess your attitudes regarding MI.**

|  | **Strongly Disagree** | **Disagree** | **Neutral** | **Agree** | **Strongly Agree** |
| --- | --- | --- | --- | --- | --- |
| As a pediatrician, I feel it is my responsibility to address my patients’ substance use. |  |  |  |  |  |
| It is important that I receive training on caring for patients with substance use. |  |  |  |  |  |
| I feel comfortable using the motivational interviewing framework to counsel a patient who screens positive for substance use. |  |  |  |  |  |

**The following questions will assess your behaviors regarding MI.**

|  | **Strongly Disagree** | **Disagree** | **Neutral** | **Agree** | **Strongly Agree** |
| --- | --- | --- | --- | --- | --- |
| I know how to counsel when someone screens positive. |  |  |  |  |  |
| I know how to refer a patient for treatment when they screen positive for substance use. |  |  |  |  |  |
