# Supplementary material for: Motivational Interviewing for Pediatric Residents: A Case-Based Workshop on Adolescent Substance Use
Source: MedEdPORTAL. 2026 Oct 22;22:11641. doi: 10.15766/mep_2374-8265.11641 (PMC13687990; doi:10.15766/mep_2374-8265.11641)
Supplement: Supplementary file 1 [file mep_2374-8265.11641-s001.zip › C. Case 1 - Cannabis HPI and SHADESS.docx]

**Using Case 1:**
The following document is for the participants. Participants will be divided into groups of three and rotate through the roles of provider, patient, and observer. All three participants should review medical background and inpatient SHADESS (adolescent social history framework: Strengths, Home, Activities, Drug, Emotions, Sexuality, Suicidality/Safety) assessment of case 1 prior to starting the role-play task. The observer should tally the number of motivational interviewing techniques they observe during the role-play on the checklist Appendix D (Case 1 and Case 2 Observer Checklist). Before starting the role-play, the provider should choose two of the skills on the checklist to practice. After participants read the case, the “provider” and “patient” will engage in a 5-minute patient encounter embracing “change talk.” They can reference Appendix A for example statements (ASAM Handout – Evoking Change Talk). For the next 5 minutes, they will reassign all three roles and engage in a 5-minute patient encounter that involved “discord” and “sustain talk” and another person will be the inpatient provider. hey can reference Appendix B for example statements (ASAM Handout- Responding to Sustain Talk).

Case 1 – Cannabis Use

Mary is a 15-year-old patient admitted to General Pediatrics for uncontrolled vomiting.

Below is a summary of her case:

ED Course

She was in her usual state of health until 2 days prior to presentation, when she developed nausea and vomiting. She has been vomiting 10-15 times a day with associated sweating and diffuse, intermittent 4/10 abdominal pain. ROS is negative for fever, rash, diarrhea, or respiratory symptoms. She tried Tylenol at home without relief. Hot showers provide temporary relief. Given that her symptoms have not improved, she presented to the ED.

Her oral intake has been inadequate, and she notes decreased urine output. There have not been any known sick contacts. She endorses smoking cannabis daily. This is her third presentation for similar symptoms in the last 6 months.

In the CHOP ED, her vital signs were initially Temp: 37.4 C HR: 124, RR: 18, and BP: 100/72. SpO2 was 100% on RA. Physical exam was notable for dry mucous membranes and mild diffuse tenderness to palpation on abdominal exam without involuntary guarding or rebound.

Labs were notable for elevated BUN, mild leukocytosis, and normal lipase and liver enzymes. Abdominal radiograph and abdominal ultrasound were unremarkable. UDS was positive for cannabinoids. She was given a bolus of normal saline and 2 doses of Zofran. Given continued vomiting, she was started on continuous fluids and admitted to the floor for further management.

Floor Course:

While on the floor, after discussing a broad differential, including potential gastrointestinal, metabolic, endocrinologic, and CNS conditions that can present with vomiting and abdominal pain, it is ultimately concluded that Cannabinoid Hyperemesis Syndrome is the most likely diagnosis. She improves with time while on intravenous hydration therapy, anti-emetics, and topical capsaicin application. You complete a SHADSSS (see document).

Task:

In groups, have a conversation with Mary for ~10 minutes, practicing the skills of motivational interviewing while assessing Mary’s readiness to change her cannabis use behaviors.

For the first 5 minutes, one person will role-play as Mary while embracing “change talk” and another person will be the inpatient provider.

For the next 5 minutes, assign new roles. One person will role-play as Mary while utilizing “discord” and “sustain talk,” and another person will be the inpatient provider.

Everyone should have an observer checklist and the ASAM handouts to help guide their roleplay.

If you are the provider, please choose two of the skills on the checklist that you particularly want to practice.

The observer’s role is to tally the number of motivational interviewing techniques they observe to help facilitate the debrief.

Example statements as Mary for “Change Talk”

Preparatory talk:

- Desire: I want, I wish, I would like to, It’d be nice if
- Ability: I can, I could, I am able to
- Reasons
- Need: I need to, I have to, I must
- Commitment: I am determined to, I am prepared to, I intend to
- Action: I am ready to
- Taking Steps: I stopped, I got rid of

Example statements as Mary for “Sustain Talk” and Discord

- My smoking is not that bad.
- It really isn’t that bad for me
- It’s the only thing that makes me feel normal
- Yup, yeah, sure, okay

Patient: Mary Jane

**Social History**

School:

Grade/ School Name/ Career Goals: Eagleview High School

Academic Performance: no concerns

School-Based Services: none

Bullying:

Do you feel that you are being bullied? no

Have there been times when you bullied others? no

Home: No concerns, gets along well with parents and older brother

Activities: Likes to spend time with friends. Enjoys music and social media. She used to be on the track team but has pulled out due to her recurrent vomiting episodes.

ETOH / Drug Use: Drugs: Smoking Marijuana daily. She gets marijuana from one of the seniors at school.

Emotional Well Being: Feels anxious at times mainly about fitting in at school.

Sexual Health: Not sexually active or thinking about becoming sexually active at present. Identifies as cis gender female and is unsure whether she is straight or bisexual but is continuing to explore this.

Safety:

Violence/Fighting: no concerns
