# Supplementary material for: Motivational Interviewing for Pediatric Residents: A Case-Based Workshop on Adolescent Substance Use
Source: MedEdPORTAL. 2026 Oct 22;22:11641. doi: 10.15766/mep_2374-8265.11641 (PMC13687990; doi:10.15766/mep_2374-8265.11641)
Supplement: Supplementary file 1 [file mep_2374-8265.11641-s001.zip › D. Case 2 - Vaping HPI and AHQ.docx]

**Using Case 2:**
The following document is for the participants. Participants will be divided into groups of three and rotate through the roles of provider, patient, and observer. All three participants should review medical background and Adolescent Health Questionnaire of case 2 prior to starting the role-play task. The observer should tally the number of motivational interviewing techniques they observe during the role-play on the checklist Appendix D (Case 1 and Case 2 Observer Checklist). Before starting the role-play, the provider should choose two of the skills on the checklist to practice. After participants read the case, the “provider” and “patient” will engage in a 5-minute patient encounter embracing “change talk.” They can reference Appendix A for example statements (ASAM Handout – Evoking Change Talk). For the next 5 minutes, they will reassign all three roles and engage in a 5-minute patient encounter that involved “discord” and “sustain talk” and another person will be the inpatient provider. hey can reference Appendix B for example statements (ASAM Handout- Responding to Sustain Talk).

Case 2 – Vaping

Nico is a 17-year-old patient who you are seeing in the clinic for a routine well child visit. You have met him once before for his 15-year-old visit. Both of his parents came with him today. You take a minute to review his Adolescent Health Questionnaire (AHQ) screener (see document), and you notice that he states he is vaping nearly every day, which was not the case at your last visit together. You are unsure if his parents are aware of his vaping.

Task:

In groups, have a conversation with Nico for ~10 minutes, practicing the skills of motivational interviewing while assessing Nico’s readiness to change his vaping.

For the first 5 minutes, one person will role-play as Nico, embracing “change talk,” while another person will be the primary care provider.

For the next 5 minutes, assign new roles. One person will roleplay as Nico while utilizing “discord” and “sustain talk” and another person will be the primary care provider

Everyone should have an observer checklist and the ASAM handouts to help guide their role-play.

If you are the provider, please choose two of the skills on the checklist that you particularly want to practice.

While debriefing, consider how you would involve Nico’s parents during the encounter.

Example statements as Nico for “Change Talk”

Preparatory talk:

- Desire: I want, I wish, I would like to, It’d be nice if
- Ability: I can, I could, I am able to
- Reasons
- Need: I need to, I have to, I must
- Commitment: I am determined to, I am prepared to, I intend to
- Action: I am ready to
- Taking Steps: I stopped, I got rid of

Example statements as Nico for “Sustain Talk” and Discord

- My vaping is not that bad.
- It really isn’t that bad for me
- It’s the only thing that makes me feel normal
- Yup, yeah, sure, okay

WELL CN ADOLESCENT HEALTH QUESTIONNAIRE

These questions will help us to get to know you better. Choose the answer that best describes what you feel or do. Your answers will be seen only by your healthcare provider and his/her assistant. Your responses will remain private/confidential, unless there are concerns you may be hurting yourself or others.

| ----------STRENGTHS--------------- | |
| --- | --- |
| Do your parent(s) or guardian(s) talk with you about your strengths (like working hard, using your talents or skills, etc.)? | Yes |
| ----------NUTRITION AND ACTIVITY--------------- | |
| Do you eat 5 or more helpings of fruits and vegetables each day? | **No!** |
| Do you drink milk and eat yogurt, cheese, or other calcium-rich foods (such as dark-green leafy vegetables, or calcium-fortified orange juice or cereal) at least 3 times each day? | Yes |
| Do you eat iron-rich food (such as meat, fish, spinach or beans) at least 5 times each week? | Yes |
| Do you drink more than 1 soda or juice drink each day? | **Yes!** |
| During the past 7 days, on how many days were you physically active for a total of at least 60 minutes per day | 3 days |
| Do you watch TV, play video games, or spend time on the computer for more than 2 hours per day (not including computer time for homework)? | No |
| Do you have any concerns or questions about the size or shape of your body, or your physical appearance? | No |
| In the past year have you tried to lose weight or control your weight by vomiting, taking diet pills or laxatives, or starving yourself? | No |
| Do you eat meals together as a family? | **No!** |
| ----------SCHOOL--------------- | |
| Are you or your parents worried about your grades? | No |
| Do you receive extra help at school for a learning problem? | No |
| Have you been told you have a learning problem, or do you think you have learning problem? | No |
| Have you been suspended from school this year? | No |
| Do you feel that you are being bullied? | No |
| Have there been times when you bullied others? | No |
| ----------FAMILY AND FRIENDS--------------- | |
| Do you know at least 1 person who you can talk to about problems? | Yes |
| Do you think that your parent(s) or guardian(s) usually listen to you and take your feelings seriously? | Yes |
| Have you ever thought seriously about running away from home? | No |
| ----------WEAPONS, VIOLENCE AND SAFETY--------------- | |
| Do you or anyone you know and/or live with carry a gun, knife, or other weapon? | No |
| Have you ever been in a physical fight where you or someone else got hurt? | No |
| Have you ever been in trouble with the police? | No |
| Are you worried about violence or your safety? | No |
| Do you always wear a seatbelt when you ride in a car, truck, or van? | Yes |
| ----------TOBACCO--------------- | |
| During the past 12 months, on how many days did you use a Juul, e-cigarette, or other vaping device (such as tanks or mods) or use other tobacco products, like cigarettes or little cigars? Put “0” if none. | **Nearly every day** |
| ----------ALCOHOL AND OTHER DRUGS--------------- |  |
| During the past 12 months, on how many days did you: Drink more than a few sips of beer, wine, or any drink containing alcohol? Put “0” if none. | 0 |
| Use any marijuana (weed, oil, or hash, by smoking, vaping, or in food) or “synthetic marijuana” (like “K2,” “Spice”) or “vaping” THC oil? Put “0” if none. | 0 |
| Use anything else to get high (like other illegal drugs, prescription or over-the-counter medications, and things that you sniff, huff, or vape)? Put “0” if none. | 0 |
| ----------ADOLESCENT DEVELOPMENT--------------- | |
| Are you dating someone or in a relationship? | No |
| Do you think you may be gay, lesbian, or bisexual? | No |
| Do you think of yourself as: (check all that apply) (no score – just informational)  Male Female Transgender male Transgender female Non-binary Other | Male |
| What was your sex at birth? | Male |
| Sex or sexual intercourse can mean different things to different people and people can have sex with others of the same or different gender. In your entire life, have you had any kind of sexual contact with anyone? | No |
| Would you like to speak with someone about STD or HIV testing today? | No |
| Would you like to speak with someone about methods to prevent pregnancy? | No |
| Do you want information about skills to manage your own health and health care? | **Yes!** |
| Do you want information to understand the changes in health care that happen at age 18? | **Yes!** |
