# Supplementary material for: Motivational Interviewing for Pediatric Residents: A Case-Based Workshop on Adolescent Substance Use
Source: MedEdPORTAL. 2026 Oct 22;22:11641. doi: 10.15766/mep_2374-8265.11641 (PMC13687990; doi:10.15766/mep_2374-8265.11641)
Supplement: Supplementary file 1 [file mep_2374-8265.11641-s001.zip › E. ASAM Handout - Responding to Sustain Talk.pdf]

Using the ASAM Handouts:
The following document should be used by the participants as reference tools during both case-based role-play cases. It will be most useful to the participant in the role of the inpatient provider. One of each handout can be printed and provided to each group or a QR code linking to this PDF can be inserted into the slides of Appendix A (MI Didactic Slides).

ASAM
MOTIVATIONAL INTERVIEWING

Eight Strategies for Responding to Sustain Talk

Sustain talk is normal and is part of ambivalence. The goal is to avoid reinforcing and amplifying sustain talk while trying to pivot back to change talk.

Simple Reflection

A simple reflection is a repeat or rephrasing of what the patient has just said. When a simple reflection is used in response to sustain talk, we are helping the patient understand that we hear what they are saying.

Example:
Patient: I have no intentions to quit drinking.
Clinician: Alcohol is something you never see yourself changing.

Amplified Reflection

An amplified reflection exaggerates or increases the magnitude of what the patient has said. This is meant to encourage the patient to correct or argue against what was said in order to elicit change talk.

Example:
Patient: I don't have any problems with marijuana.
Clinician: Marijuana hasn't caused you any problems.

Double-Sided Reflection

A double-sided reflection combines sustain talk with some previously stated change talk, in order to shift the focus of the conversation. Make sure to end the reflection with the change talk as a way to continue the conversation towards the target behavior.

Example:
Patient: I know smoking is bad for me, but it calms me down.
Clinician: On one hand, smoking makes you feel calm, and on the other hand, you're concerned about your health.

Coming Alongside (agreeing without reserve)

Coming alongside or agreeing without reserve is a strategy where you join in the patient's sustain talk, perhaps even adding in a little bit of amplification, as a way to trigger some change talk.

Example:
Patient: Opioids are really important to me.
Clinician: Opioids are really important to you. I hear you.

Reframing (suggesting a different meaning or perspective)

Reframing is a strategy where you validate the patient's perspective and then suggest a different perspective or meaning to what was just said.

Example:

Patient: I've tried to stop before, but I only made it three months.
Clinician: You stopped smoking for three months; you did that on your own.

Agree With a Twist (reflection + reframe)

Agree with a twist is a strategy where you acknowledge and agree with what the patient is saying, then reframing or "twist" what was said slightly. It is important that when presenting the "twist" (or reframe) that it is done in a calm manner so that there is no emphasis or inflection on the statement that the patient could perceive as adversarial

Example:

Patient: People always think they know what's best for me.
Clinician: People act like you don't know what's best for you. No one will make better decisions for you than you will.

Emphasizing Autonomy

Emphasizing autonomy is a strategy where you emphasize to the patient that it is ultimately their decision whether they will make a change or not. You are honoring the patient's ability (and autonomy) to make their own choices. This technique is helpful in avoiding discord when a patient might feel as if they are being coerced towards change.

Example:

Patient: I am not ready to stop drinking.
Clinician: And that's your choice to make. Only you can choose to stop.

Running Head Start

Running Head Start is a strategic response that resembles a decisional balance (pros and cons) but is still in favor of change. It should be used only when there is little to no change talk happening. Start by exploring the sustain talk, listen to the cons first to get access to the pros of change. Hear out the main motivations for reluctance and then ask: "What is the downside of not changing?"

Caution: This is not a routine procedure in motivational Interviewing since in not every instance do we routinely start with emphasizing sustain talk. This is indicated when we haven't been able to successfully evoke change talk initially, and when exploring sustain talk first might yield subsequent sustain talk.

Example:

Clinician: You like to have fun and only take pills when you are with your friends.
Patient: Exactly.
Clinician: What else do you like about the pills.

Patient: They feel good.
Clinician: You like the way the pills make you feel.
Patient: It's great.
Clinician: What else did I miss about the positives of taking these pills?
Patient: That's pretty much it.
Clinician: What might be any disadvantages of taking these pills?
