# Supplementary material for: Motivational Interviewing for Pediatric Residents: A Case-Based Workshop on Adolescent Substance Use
Source: MedEdPORTAL. 2026 Oct 22;22:11641. doi: 10.15766/mep_2374-8265.11641 (PMC13687990; doi:10.15766/mep_2374-8265.11641)
Supplement: Supplementary file 1 [file mep_2374-8265.11641-s001.zip › F. ASAM Handout - Evoking Change Talk.pdf]

Using the ASAM Handouts:
The following document should be used by the participants as reference tools during both case-based role-play cases. It will be most useful to the participant in the role of the inpatient provider. One of each handout can be printed and provided to each group or a QR code linking to this PDF can be inserted into the slides of Appendix A (MI Didactic Slides).

ASAM
MOTIVATIONAL INTERVIEWING

Evoking Change Talk to Support Patient Motivation: *Follow These Steps*

Step 1: *Ask evocative, open-ended questions.*

The simplest and most direct way of evoking change talk is to ask for it:

- **Ask open-ended questions** for which change talk is the answer:
  - *Examples:*

    - Why would you want to make this change? (Desire)

    - How might you go about it to succeed? (Ability)

    - What are the three best reasons for you to do it? (Reasons)

    - How important is it for you to make this change? (Need)

    - So what do you think you'll do? (Commitment)

- **Ask for examples**:
  - *Examples:*

    - When was the last time that happened?

    - Describe a specific example of when this happens.

    - What else?

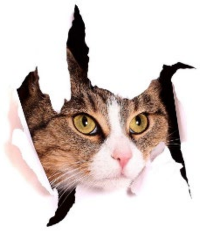

- ***D.A.R.N. C.A.T.S..:*** Consider questions that signal if the patient can change if the patient sees the need for change: Initially, consider signals of ***preparatory change talk***. Next, consider signals of ***mobilizing change talk.***
- If you're hearing a lot more preparatory change talk, but not enough mobilizing then you can think about ways in which you can ask for questions about steps that the patient has taken in the past, or is taking already, that will create greater change to give more mobilizing change talk.

Step 2: ***Use the readiness rulers to explore the importance and confidence patients associate with changing behavior.***

Explore the patient's views on the ***importance*** of making a behavior change as well as their ***confidence*** in their ability to make that change using a scale of 1 to 10.

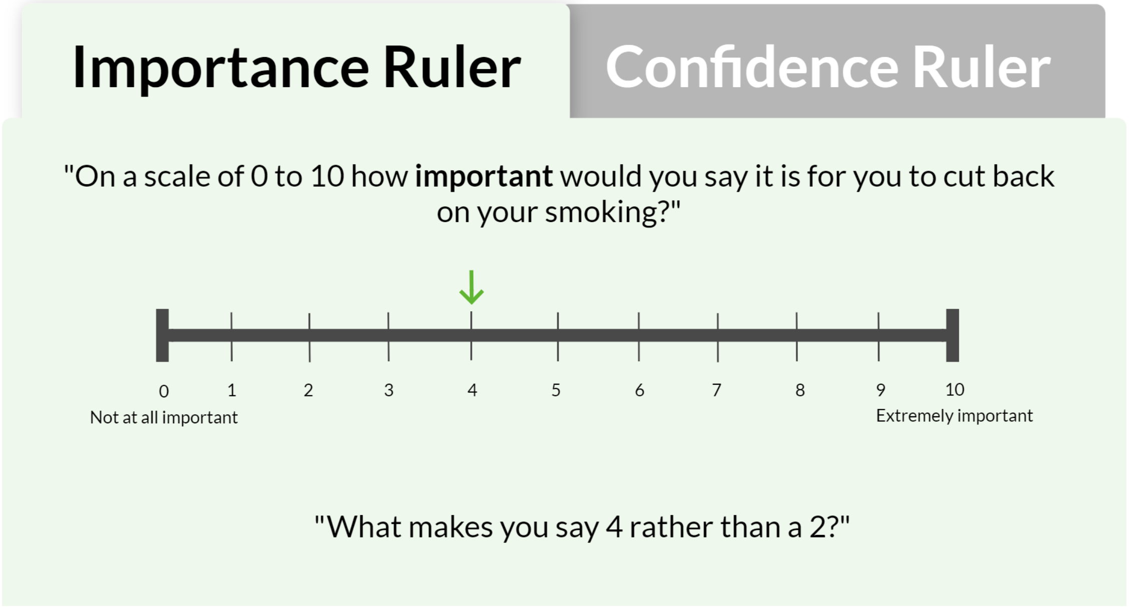

Importance	*Confidence*
Ask:<br>• “On a scale of 1 to 10, how **important** is it for you to change [behavior]?<br>Follow up:<br>• What makes you say [patient’s #], rather than [lower #]?<br>• What would it take for you to go from a [patient’s #] to [higher #]?	Ask:<br>• “On a scale of 1 to 10, how **confident** are you that you can change [behavior]?

Step 3: *Explore decisional balance with the patient.*

Decisional balance is a tool from other approaches that can be used in the service of eliciting change talk. In doing a decisional balance, consider the pros and cons of both making the change and staying the same.

PROs:

- What are the advantages if you were to stop [insert behavior]?

- What are the advantages if you were to continue [insert behavior]?

CONs:

- What are the disadvantages of continuing [insert behavior]?

- What are the disadvantages of stopping [insert behavior]?

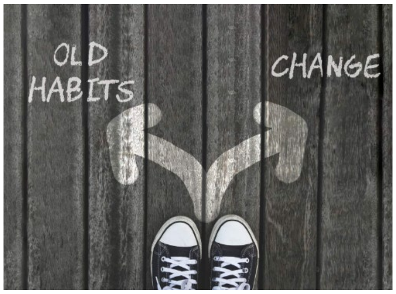

Step 4: *Ask for elaboration when a change topic comes up.*

As patients clarify their thoughts and feelings, you obtain more information and can likely explore topics that are meaningful to the patient.

- **When a change talk topic comes up**:
    - *Examples:*

      - In what ways does [behavior] impact you? /How does [behavior] impact you?

      - How do you see this happening?

      - What have you changed in the past that you can relate to this issue?

      - Tell me an example of what happens when…

Step 5: *Look back. /Look forward.*

- Elicit past successes and strengths and anticipated future advantages of making this change.
    - *Looking Back Examples:*

      - How were you able to stop drinking in the past?

      - Before your drinking became a problem, how were things better/different?

    - *Looking Forward Examples:*

      - What would be different if you were 100% successful in making changes?

      - How would you like your life to be different one week from now?

      - What might happen if you don't make any changes?

Step 6: *Explore goals and values.*

- Understand their motivations for change so that you can work to enhance it.
    - *Examples:*

      - What sort of person [parent/daughter/son/wife/partner] do you want to be?

Summary: *Clinicians use these strategies to evoke change talk. Change talk predicts subsequent behavior change.*

Adapted from Motivational Interviewing Network of Trainers (MINT) Resources:
https://motivationalinterviewing.org/motivational-interviewing-resources
